# Supplementary material for: Foulant Identification and Performance Evaluation of Antiscalants in Increasing the Recovery of a Reverse Osmosis System Treating Anaerobic Groundwater
Source: Membranes (Basel). 2022 Mar 2;12(3):290. doi: 10.3390/membranes12030290 (PMC8950045; doi:10.3390/membranes12030290)
Supplement: Supplementary file 1 [file membranes-12-00290-s001.zip › membranes-1603471-supplementary.pdf]

membranes

MDPI

Supplementary material for:

Foulant Identification and Performance Evaluation of Antiscalants in Increasing the Recovery of a Reverse Osmosis System Treating Anaerobic Groundwater

M. Nasir Mangal [1,2,*], Sergio G. Salinas-Rodriguez [1], Jos Dusseldorp [3], Bastiaan Blankert [3,4], Victor A. Yangali-Quintanilla [5], Antoine J. B. Kemperman [2], Jan C. Schippers [1], Walter G. J. van der Meer [2,3] and Maria D. Kennedy [1,6]

1 IHE Delft Institute for Water Education, Water Supply, Sanitation and Environmental Engineering Department, Westvest 7, 2611 AX Delft, The Netherlands; s.salinas@un-ihe.org (S.G.S.-R.); jancschippers@gmail.com (J.C.S.); m.kennedy@un-ihe.org (M.D.K.)
2 Faculty of Science and Technology, University of Twente, P.O. Box 217, 7500 AE Enschede, The Netherlands; a.j.b.kemperman@utwente.nl (A.J.B.K.); w.g.j.vandermeer@utwente.nl (W.G.J.v.d.M.)
3 Oasen Drinkwater, Nieuwe Gouwe O.Z. 3, 2801 SB Gouda, The Netherlands; jos.dusseldorp@oasen.nl
4 Water Desalination and Reuse Center (WDRC), Biological and Environmental Science and Engineering Division (BESE), King Abdullah University of Science and Technology (KAUST), Thuwal 23955-6900, Saudi Arabia; bastiaan.blankert@kaust.edu.sa
5 Grundfos Holding A/S, Water Solutions, Poul Due Jensens Vej 7, 8850 Bjerringbro, Denmark; vyangali@grundfos.com
6 Faculty of Civil Engineering, Delft University of Technology, Stevinweg 1, 2628 CN Delft, The Netherlands
* Correspondence: m.mangal@un-ihe.org

Equations:

The equations S1–S6 for calculating normalized permeability are obtained from the membrane manufacturer (Hydranautics) and the ASTM standard practice for standardizing RO performance data (Designation: D4516–00).

$$K_w = \frac{Q_P}{NDP \times A} \times \frac{TCF_r}{TCF_t} \qquad \text{Eq. S1}$$

Where: Q_p = permeate flow (L/h); NDP = net driving pressure (bar); A = membrane area (m^2); TCF_r = temperature correction factor at reference conditions (25 °C) which is equal to 1; TCF_t = temperature correction factor at time t.

$$NDP = P_f - \frac{\Delta P_{fc}}{2} - P_p - \pi_{fc} + \pi_p \qquad \text{Eq. S2}$$

Where: P_f = feed pressure (bar); ΔP_{fc} = pressure drop (bar); P_p = permeate pressure (bar); π_{fc} = feed-concentrate osmotic pressure (bar); π_p = permeate osmotic pressure (bar).

$$\pi_{fc} = 0.002654 \times C_{fc} \times \frac{(T + 273)}{(1000 - \frac{C_{fc}}{1000})} \qquad \text{Eq. S3}$$

$$\pi_p = 0.002654 \times C_p \times \frac{(T + 273)}{(1000 - \frac{C_p}{1000})} \qquad \text{Eq. S4}$$

$$C_{fc} = \frac{C_f + C_c}{2} \qquad \text{Eq. S5}$$

Where: C_f = TDS of the feed (mg/L); C_c = TDS of the concentrate (mg/L); C_p = TDS of the permeate (mg/L); T = Temperature (°C)

$$TCF = e^{2700 \times \left(\frac{1}{298} - \frac{1}{273 + T}\right)} \qquad \text{Eq. S6}$$

Figures:

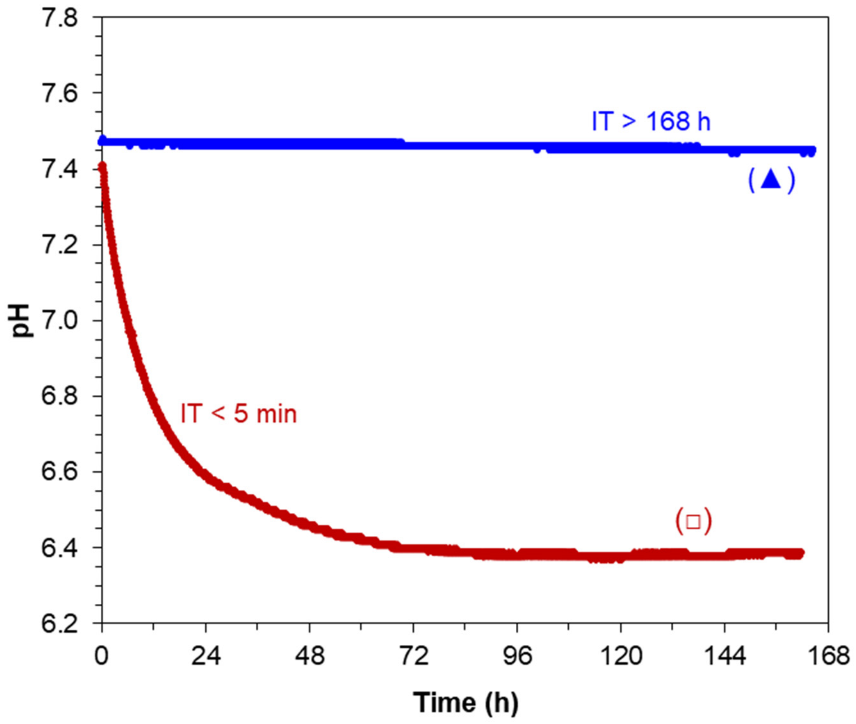

Figure S1. Induction time (IT) of the (▲) real RO concentrate at 85 % recovery without antiscalant, and (□) artificial RO concentrate of 85 % recovery without antiscalant.

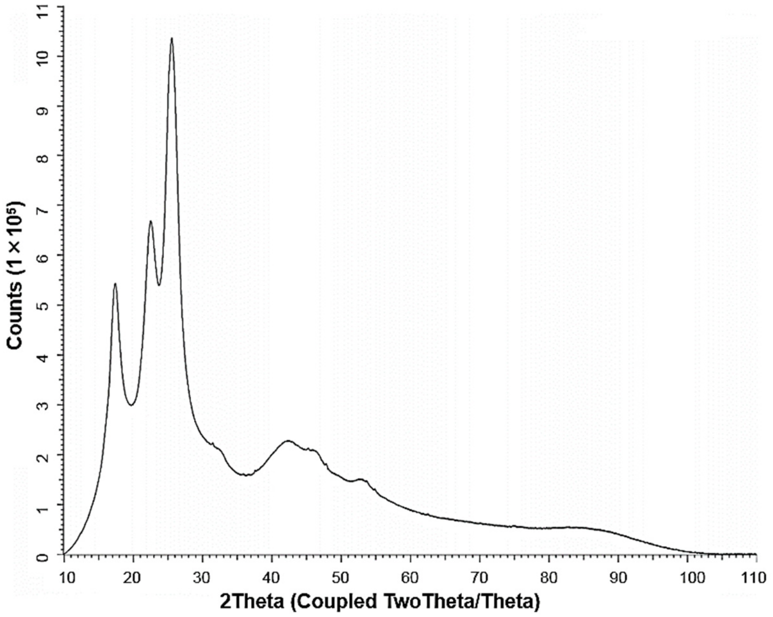

Figure S2. XRD analysis of the fouled membrane (tail element) of the 3rd stage.

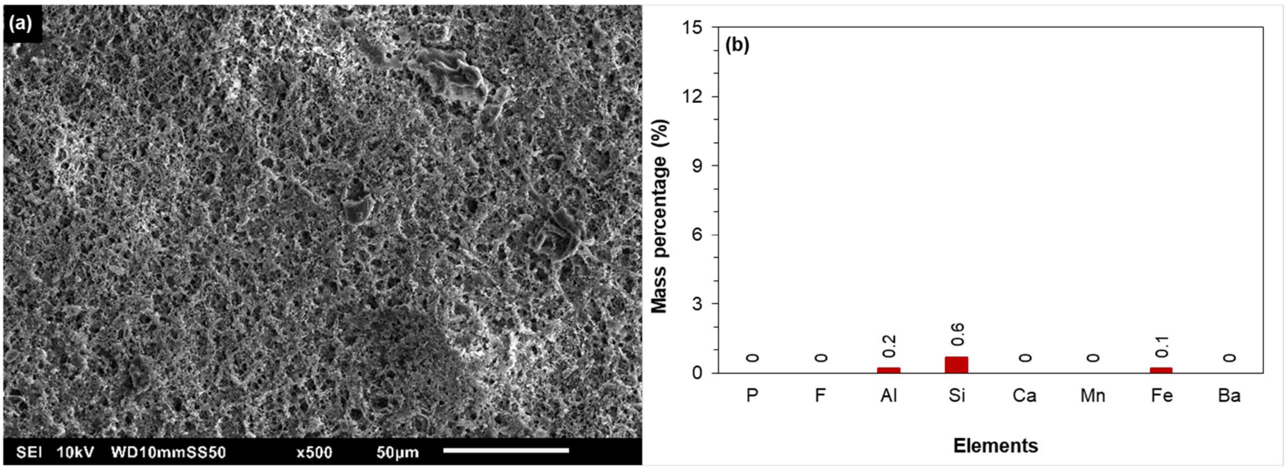

Figure S3. (a) SEM image and (b) EDX analysis of the 0.45 μm filter after filtration of the 0.05 M HCl solution (of the tail element of the 3rd stage).

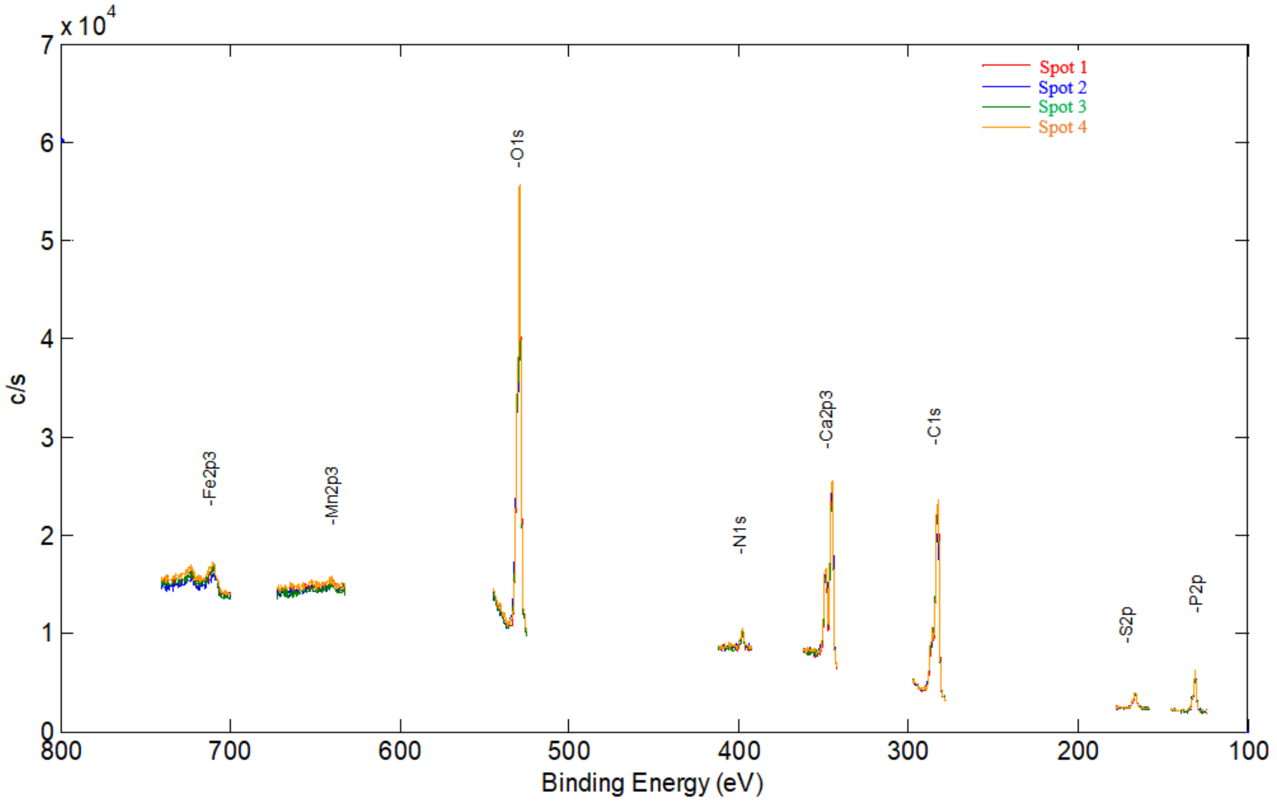

Figure S4. (a) XPS analysis of the fouled membrane (tail element) of the 3rd stage.

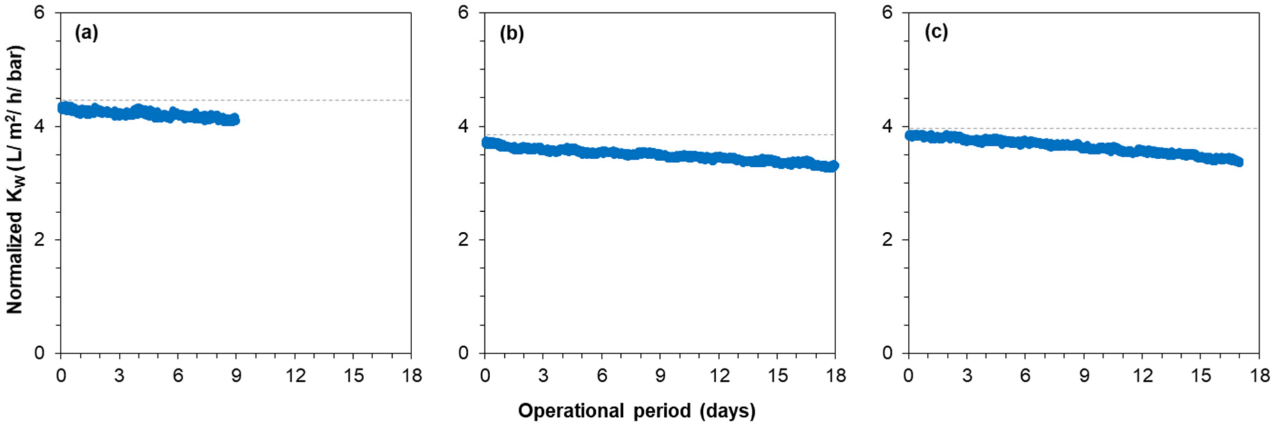

Figure S5. a) Average normalized permeability of the last stage of the RO unit at 83% recovery (a) without antiscalant, (b) with 2.0 mg/L AS–1, and (c) with 2.0 mg/L AS–2.

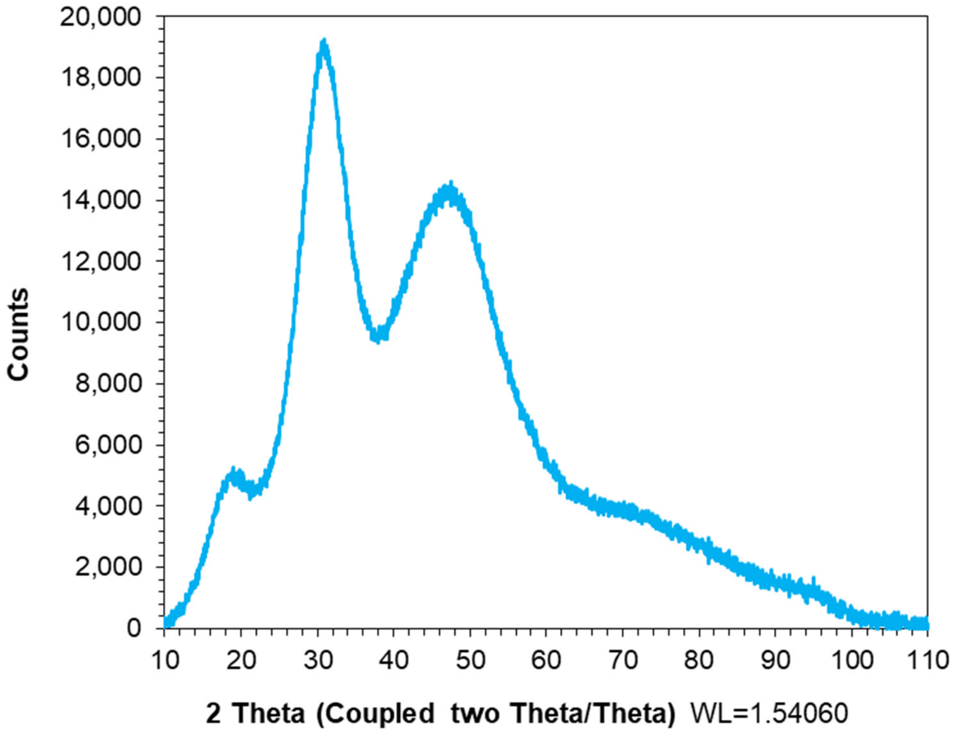

Figure S6. XRD analysis of the small RO element fouled with calcium phosphate in once-through lab-scale RO measurements.
